# Novel Therapeutic Strategies for Ovarian Cancer Stem Cells

**DOI:** 10.3389/fonc.2020.00319

**Published:** 2020-03-17

**Authors:** Nastassja Terraneo, Francis Jacob, Anna Dubrovska, Jürgen Grünberg

**Affiliations:** ^1^Center for Radiopharmaceutical Sciences ETH-PSI-USZ, Paul Scherrer Institute, Villigen, Switzerland; ^2^Ovarian Cancer Research, Department of Biomedicine, University Hospital Basel and University of Basel, Basel, Switzerland; ^3^OncoRay-National Center for Radiation Research in Oncology, Faculty of Medicine and University Hospital Carl Gustav Carus, Technische Universität Dresden, Dresden, Germany; ^4^German Cancer Consortium (DKTK), Partner Site Dresden, Dresden, Germany; ^5^German Cancer Research Center (DKFZ), Heidelberg, Germany; ^6^Helmholtz-Zentrum Dresden-Rossendorf, Institute of Radiooncology-OncoRay, Dresden, Germany

**Keywords:** ovarian cancer, cancer stem cells, epithelial-to-mesenchymal transition, L1CAM, radioimmunotherapy, Auger electron and alpha particle emitters, therapeutic strategies

## Abstract

Ovarian cancer (OC) is one of the most lethal gynecologic malignancies. Due to the lack of specific symptoms and screening methods, this disease is usually diagnosed only at an advanced and metastatic stage. The gold-standard treatment for OC patients consists of debulking surgery followed by taxane combined with platinum-based chemotherapy. Most patients show complete clinical remission after first-line therapy, but the majority of them ultimately relapse, developing radio- and chemoresistant tumors. It is now proposed that the cause of recurrence and reduced therapy efficacy is the presence of small populations of cancer stem cells (CSCs). These cells are usually resistant against conventional cancer therapies and for this reason, effective targeted therapies for the complete eradication of CSCs are urgently needed. In this review article, we highlight the mechanisms of CSC therapy resistance, epithelial-to-mesenchymal transition, stemness, and novel therapeutic strategies for ovarian CSCs.

## Introduction

### Ovarian Cancer

Ovarian cancer (OC), which is a generic term for several different malignant tumors, is one of the most frequent cancer types in females (1). OC is the leading cause of cancer death among gynecological malignancies, and according to estimates, in 2019 ~22,530 new cases of OC will be diagnosed and 13,980 OC-related deaths will occur in the U.S. (2). About 75% of all ovarian tumors and 90–95% of ovarian malignancies are epithelial ovarian cancer (EOC) (3) of different origin (4). The 5-year survival rate of OC varies from 30 to 92%, depending on the dissemination of the disease at the time of diagnosis (5). This type of cancer usually shows non-specific symptoms during the early development (e.g., abdominal distension and pain, loss of appetite, or increased urinary frequency) and currently there are still no reliable early screening strategies available (6). The most common sign of advanced disease is abdominal swelling due to ascitic fluids accumulation (7). OC is consisting of different histological subtypes with distinctive molecular genetic features, clinical presentations, and prognostic outcomes. In 2014, the new WHO criteria recognized five principal epithelial OC histotypes: high-grade serous (HGSC), low-grade serous (LGSC), endometrioid, clear cell, and mucinous carcinoma (8). Among them, HGSC is the most common histologic subtype of OC, with a poor 5-year survival rate of 35–40% and accounting for 70–80% of OC deaths (5, 9). Because of the lack of diagnostic methods, ~75% of patients show the metastatic spread in the peritoneal cavity and adjacent organs at diagnosis, which corresponds to FIGO stages (International Federation of Gynecology and Obstetrics stages) III and IV (10). The FIGO OC staging system was first published in 1973 and revised in 2014 (11, 12). Stage I EOC (disease is confined to one or both ovaries) is usually associated with good survival rates and surgery alone is sufficient as a therapeutic approach; unfortunately, it is quite rare since most of the patients are diagnosed only at stages III and IV (13). At stage II, tumor includes either one or both ovaries with pelvic dissemination and spread into the uterus and/or fallopian tubes. This group makes up <10% and it is considered curable, usually with chemotherapy (12). Stage III EOC tumor implicates one or both ovaries with spread to the peritoneum, lymph nodes and/or other sites outside the pelvis. The majority of OC are HGSCs and patients are diagnosed at stage III (14). Stage IV is characterized by distant metastases affecting the liver, spleen and lymph nodes and/or to other organs or tissues outside the peritoneal cavity such as the lungs and bones.

### Ovarian Cancer Treatments

Current first-line treatment regimen for OC patients comprises complete debulking surgery. The reductive tumor procedure includes hysterectomy, omentectomy, and other affected tissues possible to remove. The goal of surgery is to reduce tumor burden and minimize residual disease, which is inversely proportional to survival (15). Indeed, residual lesions smaller than 2 cm have been associated with better survival than bigger ones (16). At the same time, debulking surgery allows to precisely establish the histologic subtype of the disease and, therefore, it is very important for diagnosis. Even though surgery is the basis for OC treatment, it is rarely curative alone for patients with advanced disease and it needs to be combined with chemotherapy.

In late 1990s, two phase III clinical trials combined cisplatin (CDDP) with paclitaxel (PTX) as adjuvant treatment for advanced stage OC (17). Ever since, the combination of taxane and platinum derivatives, like CDDP and carboplatin (CBT), has been used as a standard therapeutic approach for OC patients, leading to response rate, and complete clinical remission of 60–80% (18). Nevertheless, the majority of these patients will ultimately relapse with a median progression-free survival of 18 months (19). Usually, response rates to second-line chemotherapy are proportional to treatment-free interval (20). Different combinations of chemotherapeutics have been tested to overcome chemoresistance following first-line paclitaxel-platinum treatment, but clinical responses are short-lived and led to only minor survival improvements for patients with chemoresistant tumors (21). So far, radiation therapy (RT) has played a minor role in ovarian cancer. Abdominopelvic RT was associated with serious side effects and poor therapeutic efficacy for most of the patients (22, 23). Acute toxicity was most commonly due to cramps, diarrhea, nausea, vomiting and more severe myelosuppression, whereas long-term toxicity was associated with bowel obstruction (23, 24). The poor therapeutic effect was due to the limitation of dosage that causes toxicity to radiosensitive organs such as blood building system or kidney. Maybe new approaches in radiotherapy could lead to wider use of RT in OC [reviewed in Fields et al. (25) and Iorio et al. (26)]. Likewise, the use of radiolabeled antibodies for the management of advanced ovarian cancer after cytoreductive surgery and chemotherapy is limited and of no success so far. Antibodies directed to CA-125 (mAb OC-125) and MUC1 (mAb HMFG1) antigens labeled with iodine-131 or yttrium-90, respectively showed little or no therapeutic benefit in ovarian cancer patients (27, 28). Different reasons for the treatment failure were discussed. The dose of radiation may have been too low because of insufficient binding of the antibody or the lack of antigen expression in residual micrometastases. Furthermore, yttrium-90 is not the ideal isotope for irradiation of small tumor nodules. No pharmacokinetics was performed, and it is possible that there was limited systemic exposure to the intact radioimmunoconjugate. In addition, the anti-MUC1 antibody HMFG1 is not actively internalized into target tumor cells. In contrast to RT, radioimmunotherapy (RIT) is a targeted therapy were the antibody brings the radiation to the tumor site also to disseminated metastases. Non-specific irradiation of healthy tissue is normally low due to the clearing of the mAb from the blood. The dose-limiting organ is the bone marrow [reviewed in Larson et al. (29)]. New promising RIT approaches for the treatment of OC will be discussed during this report.

The better understanding of tumor biology and chemoresistance over the past years supported the development of molecular targeted therapies, improving survival and increasing the quality of life in OC patients. Many different inhibitors, such as tyrosine kinase inhibitors (30) and monoclonal antibodies (mAbs) targeting multiple crucial cancer pathways, including angiogenesis, cell survival, cell growth, metastasis formation and DNA repair, are currently tested in clinical trials (31). The most promising investigational agents include vascular endothelial growth factor (VEGF)-specific inhibitors and poly (ADP-ribose) polymerase inhibitors (PARPi). Bevacizumab (Avastin®, Genentech, Inc.), a recombinant humanized mAb against VEGF, blocks angiogenesis, enhancing the efficacy of standard therapy. In 2004, Bevacizumab has been clinically approved in the U.S. as the first angiogenesis inhibitor for colon cancer (32). In 2018, based on phase III GOG-0218 clinical study (NCT00262847), the FDA approved its use in combination with CBT and PTX, followed by single-agent bevacizumab for the treatment of patients with advanced (stage III or IV) ovarian epithelial, primary peritoneal or fallopian tube cancer after initial surgery (33). PARP enzymes are involved in different cellular functions, including DNA single-strand break (SSB) repair through base-excision repair by PARP1 (34). The first PARPi approved in the clinic was Olaparib (AZD2281, Ku-0059436 trade name Lynparza), an orally administered drug (35). In 2014, based on phase III SOLO-2 (NCT01874353) and phase II Study 19 (NCT00753545) clinical trials, Olaparib obtained an accelerated FDA approval as maintenance treatment for patients with a recurrent ovarian epithelial, fallopian tube or primary peritoneal cancer, who are in complete or partial response to platinum-based chemotherapy (35–38). In the same year, based on phase II Study 19 and phase II Study 42 (NCT01078662), the EMA authorized Olaparib as maintenance treatment for patients with platinum-sensitive, relapsed BRCA-mutated (germline or somatic) HGSC, who responded to the last platinum-based chemotherapy (38, 39). Other types of tumorigenic pathway inhibitors targeting PI3K/AKT, mTOR, Src, and FRα are still in the early phase of development (38). To date, no effective cure for OC has been found. Considering the heterogeneous nature of OC and the lack of a common deregulated pathway in most patients, individualized therapy seems to be essential to improve survival.

## Tumor Heterogeneity

**Figure 1:**
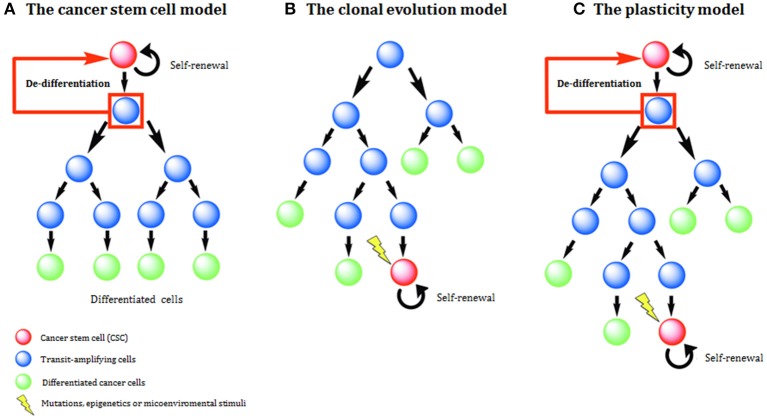
Models of tumor development and heterogeneity. **(A)** The cancer stem cell (CSC) model of tumor development. Genetic or epigenetic mutations activate stem-like programs in a single cell, generating a CSC. This CSC is able to indefinite self-renewal and/or differentiation and all derived tumor cells have a hierarchical inheritance pattern. **(B)** The clonal evolution (CE) model of tumor development. Due to the acquisition of epigenetic and genetic mutations through time, any cell might have tumorigenic potential. Tumor heterogeneity is due to the propagation of cells carrying genetic mutations. **(C)** The plasticity model highlights the plastic state of cancer stemness. Based on the model, differentiated non-tumorigenic cancer cells can potentially revert back to CSCs.

Two main models have been used to explain histological and molecular heterogeneity, a common feature of most solid tumors: the clonal evolution (CE) or stochastic model and the cancer stem cell (CSC) or hierarchical model. In recent times a third model, called the plasticity model, linking CE, and CSC models has been postulated (Figure 1).

### The Clonal Evolution or Stochastic Model

In the 1970s, with the discovery that mutations in oncogenes and tumor suppressor genes trigger most human cancers, Nowell introduced the clonal evolution (CE) concept (40). The CE model assumes that every tumor cell is biologically equivalent and potentially able to drive tumor progression (41). The majority of cancer cells have only restricted proliferative potential and tumor progression is driven by the acquisition of gene mutations and epigenetic alterations in the original clone (42, 43). The progress from early to invasive carcinoma implicates the stepwise acquisition of random mutations in specific cancer genes, leading to uncontrolled proliferation and high tumor heterogeneity (44).

### The Cancer Stem Cell or Hierarchical Model

In the early 1990s, with the introduction of new technologies such as fluorescence-activated cell sorting (FACS) and mouse xenografts assays for hematopoietic stem cells, Dick and colleagues found that tumor engraftment in acute myelogenous leukemia (AML) could only be initiated by CD34^+^/CD38- cell population (45). In 2003, Clarke et al. applied for the first time the same experimental approach to solid breast cancer (46). Using xenograft assay, they showed that as few as 100 CD44^+^/CD24^−^/low breast cancer cells were sufficient to induce tumors, in contrast to thousands of cells expressing different markers (46). Moreover, this tumorigenic population has been passaged several times and these cells were always able to induce tumors recapitulating the original tumor composition, forming both CD44^+^/CD24^−^/low tumorigenic and non-tumorigenic cells. Afterwards, similar studies on other solid tumors, such as brain, breast, prostate and colon cancer, were published (47, 48). Most studies on CSCs have been performed in a similar way. Generally, small populations of cells defined by a specific marker or marker panel and expressed in a heterogeneous way in a particular type of tumor, are isolated from cell lines or primary tumors. When transplanted into immunodeficient mice, these cells can induce tumor growth over weeks or months and to reproduce the heterogeneity of the initial tumor (49). A frequency of CSCs present in a given tumor population can be analyzed in limiting dilution assay (LDA) by transplanting increasing dilutions of single tumor cell suspensions. LDA analysis *in vivo* is the gold standard to determine the CSC frequency in a given tumor cell population (50, 51). In addition to LDA, subsequent transplantation assays provide an important information about the long-term self-renewal and tumor regeneration capacity of the tentative CSC populations (52). An emerging gene engineering technologies including applications of CRISPR-*Cas9* system for target genome editing substantially simplified generation of knockin or knockout cell and mice models. The genetically modified patient-derived organoid and mice models where a given cell population can be traced *in vivo* is an important tool to identify tumor cell of origin (53, 54). Nevertheless, because of technical issues, many theoretical and experimental details about the CSC model have remained unexplored and the frequency of CSCs in solid tumors is highly variable. Technical issues include inconstant purity of tumor cell isolation, the necessity of more solid and reliable markers and the challenges related to xenotransplant assays that offer a different environment than the original tumor niche (55).

The CSC model suggests that the origin and the progression of many cancers are driven by small subpopulations of cells with stem-like properties; however, this model does not address the question of whether tumors arise from normal stem cells. Instead, it suggests that, regardless of the cell-of-origin, many cancers are hierarchically organized in the same manner as normal tissues and CSCs share similar molecular properties to normal stem cells. In accord with this model, tumors have a hierarchical structure, with tumorigenic CSCs at the top that generate both intermediate progenitors (also called transit-amplifying cells) and terminally differentiated cells. Considering that the same CSC populations can originate from different cancer subtypes, the frequency of CSCs can highly vary among tumor types and also within the same tumor, leading to tumor heterogeneity (56). CSCs, like non-neoplastic stem cells, have extensive proliferative potential and generate the differentiated progeny that form most of the tumor mass and it is highly sensitive to cancer therapies. Additionally, these cells can remain quiescent for prolonged periods of time, which renders them unresponsive toward radiation and chemical insults, including cytotoxic drugs designed to target fast-proliferating tumor cells (57). Interestingly, recent studies have highlighted some common features (58, 59) but also many differences in stem cell programs operating in CSCs and non-neoplastic stem cells (60).

### The Plasticity Model

It is now evident that one model does not exclude the other and both might contribute to cancer development, depending on tumor type and stage (61).

In recent years, an alternative model based on cellular plasticity, which links the CE and the CSC models, has emerged (61–63). The plasticity model proposes that cancer cells in different types of tumors including OC can switch between stem cell-like and differentiated states so that some differentiated non-tumorigenic cancer cells can de-differentiate to become CSCs (64). Therefore, CSC-like phenotype is flexible and dynamic, instead of being a fixed property of tumor cells. Signaling within the tumor microenvironment (tumor niche), including oxygenation, cell-to-cell contact and secreted factors, could induce differentiated tumor cells to re-acquire stem cell-like properties (62). Additionally, radio- and chemotherapy treatments have been shown to enrich CSC subpopulations in residual tumors because of selective pressure on drug-resistant cells (65–67) and due to tumor cell plasticity (64). Even though the CSC state has high plasticity, it is of high clinical importance as a potential marker for clinical outcome and target for anti-cancer treatment (68, 69).

## Ovarian Cancer Stem Cells

Regardless of the high response rate to standard therapy, most OC patients develop recurrent chemoresistant disease (70). Recurrence is believed to be caused by the presence of residual tumor-propagating cells that cannot be completely eradicated by surgical and/or pharmacological regimens (9). Accumulating evidence suggests that among these residual cancer cells some have the key stem cell-like properties such as self-renewal and differentiation (71, 72). This small population of cells appears to form and to sustain the tumor bulk population, being responsible for disease recurrence after the first-line treatment (73). In some studies, these cells have been isolated by flow cytometry and were discovered to be enriched in a side population (SP) able to efflux the Hoechst33342 dye by cell transporters using the same mechanism with which normal cells efflux toxic drugs (74, 75). Further investigations revealed that these cells have several characteristics in common with normal tissue stem cells. In 2005, Bapat et al. were one of the first groups that characterized the presence of ovarian CSCs from patient ascites, showing tumorigenic properties of these cells (71). In addition to self-renewal and the ability to give rise to more differentiated progeny, CSCs are highly tumorigenic and display increased resistance against conventional cancer therapies (76–78). As of today, there are still considerable controversies on the OC CSCs due to the heterogeneity of CSC phenotypes, plasticity of CSC states as well as limitations of the current research methodology for CSC characterization. Nevertheless, reliable markers of OC CSCs have been successfully validated by xenograft transplantations of the serial tumor cell dilutions (limiting dilution assay) along with serial tumor transplantation and lineage-tracing assays (79–82). These analyses are currently “gold standard” assays to measure key CSC properties such as self-renewal and multipotency (52). In support of these preclinical findings, clinical significance of CSCs was recently confirmed by a number of studies demonstrated the association of CSC markers with clinicopathological parameters and clinical outcomes of OC patients (69).

### Ovarian Cancer Stem Cell Markers

The proportion of CSCs can vary depending on tumor type and in the context of OC, CSC frequency shows high interpatient variability (83). Considering CSCs resistance against conventional cancer treatments, the development of more efficient tumor therapies requires effective identification and functional characterization of these cell populations. The expression of several individuals or combined cell surface markers has been associated with CSCs (Table 1). The multiplicity of CSC markers, along with their plasticity, might pose a challenge to detect successful CSC-targeting therapeutic strategies. For this reason, it is important to select reliable molecular markers that could be used to develop new therapies.

**Table 1:** List of some putative CSC markers.

**CSC marker**	**Protein function**	**References**
CD133	Pentaspan transmembrane glycoprotein—membrane organization	(52–61)
ALDH	Detoxifying enzyme–drug resistance	(62–70)
CD44	Cell adhesion molecule—receptor for hyaluronic acid	(47, 73–77)
CD24	Mucin-like cell adhesion molecule	(78–80, 84)
CD326	Epithelial cell adhesion molecule (EpCAM)	(38, 81–83, 85)

One of the best-characterized CSC surface markers is the transmembrane glycoprotein CD133 (also known as AC133 and Prominin-1), initially identified as hematopoietic stem cell marker supporting stem cell maintenance and expansion (85, 86). CD133 localizes to plasma membrane protrusions and microvilli, indicating its role in membrane organization (86). Several recent studies revealed the role of CD133 as a positive regulator of Wnt, PI3K and EGFR signaling pathways (87, 88). However, the exact physiological function of CD133 in normal and cancer cells is still elusive. CD133 was first characterized as CSC marker in glioblastoma (89) and later it was found to be widely expressed in tumor-initiating cells of different tumors (e.g., ovarian, liver, lung, pancreatic and prostate cancer) (90). Several groups have identified the expression of CD133 in OC cells, which is connected with tumor initiation, self-renewal and chemoresistance (91, 92). Some published studies indicated the phenotypic heterogeneity and plasticity of CD133 expression (80). This finding might explain why some data did not support the link between CD133 and ovarian CSCs, showing inconsistent expression and no increased spherogenic or tumorigenic properties of CD133+ in comparison to the CD133− counterpart (93, 94). Inconsistent CD133 detection because of different immunoreactivity of primary anti-CD133 antibody clones might also account for discrepancies in the identification of CSCs (95).

The human genome encodes 19 aldehyde dehydrogenase (ALDH) enzymes implicated in detoxification of endogenous and exogenous aldehydes via NAD(P)+-dependent oxidation (96, 97). Upon chemotherapy and irradiation, these enzymes catalyze the oxidation of aldehydes (oxygen, carbon, and hydrogen) to carboxylic acids to prevent DNA damage. ALDH modulates the expression of drug transporters to efflux chemotherapeutic agents, contributing to cell-acquired drug resistance against conventional cancer therapies (98). Additionally, the ALDH protein family catalyzes the oxidation of retinoic acid, which regulates the differentiation of normal stem cells and CSCs (99). The ALDH1 subgroup is highly expressed in normal and CSCs. Among ALDH1 isozymes, ALDH1A1 is more widely expressed in CSCs of different cancer types than ALDH1A2 and ALDH1A3 (100). The study of the ALDH1 expression in 24 types of normal and six types of epithelial tumor tissues revealed that increased expression of ALDH1 was significantly associated with poor outcomes for 439 patients with serous OC (68). Furthermore, ALDH expression in combination with CD133 correlates with poor patient prognosis and characterizes an ovarian CSC population (76, 101). However, recent research suggested that ALDH is a better marker to identify ovarian CSCs than CD133, as ALDH correlates with tumorigenicity and spheroid formation (102). It is now well-known that only cancer progenitor cells have the ability to proliferate under non-differentiating and non-adherent conditions, forming 3D tumor spheres (103). These spheres are enriched by cells displaying stem cell-like properties, including the upregulation of some stem cell-specific genes, high ALDH activity, self-renewal ability along with high proliferative and differentiation properties (102, 104, 105). Consequently, sphere-forming cells display aggressive growth, migration, invasion, clonogenic survival, anchorage-independent growth, and reduced drug responsiveness *in vitro* (105).

CD44 cell transmembrane glycoprotein has been associated with several signal transduction pathways, including NANOG and EGFR-Ras-ERK (106). The main CD44 ligand is hyaluronic acid, a component of the extracellular matrix, which is positively associated with OC migration and metastatic spread (107, 108). CD44 is expressed in both normal and ovarian CSCs and it is associated with sphere-forming ability, self-renewal, chemoresistance, tumorigenicity, proliferation and invasiveness (109). Additionally, high expression of CD44 correlates with epithelial-to-mesenchymal transition (110) and with stem cell-like properties, contributing to tumor invasion, metastasis, disease recurrence and chemoresistance (111). The co-expression of CD44 with the c-Kit receptor CD117 was shown to define an ovarian subpopulation with tumor-initiating capacity (72). C-kit regulates survival, proliferation and chemoresistance of ovarian CSCs through PI3K/AKT and Wnt/β-catenin-ATP-binding cassette G2 signaling (112).

CD24 is a glycosylphosphatidylinositol-anchored membrane glycoprotein recognized as a positive or negative marker for CSCs in numerous cancer types. The low expression of CD24 combined with high CD44 (CD44+/CD24−/low) is used to identify breast CSCs (46). On the contrary, several data indicated CD24 as a positive marker for ovarian CSCs. A population of CD24+ cells isolated from OC samples and cell lines was shown to display high expression of stemness-related genes, high tumor initiation and fast tumor growth along with increased sphere-forming ability (113, 114). Besides, Davidson and colleagues demonstrated that tumor cells collected from OC peritoneal fluids exhibited higher levels of CD24 than solid tumors, suggesting an enrichment of CSCs (115).

Epithelial cell adhesion molecule (EpCAM), also called CD326, is a transmembrane glycoprotein overexpressed in many carcinomas (116). Several studies described a controversial role of EpCAM in carcinogenesis. Being an adhesion molecule, EpCAM regulates homophilic adhesion interactions, which might inhibit metastatic invasion (116). However, depending on the microenvironment, EpCAM is also able to suppress E-cadherin adhesions, supporting metastasis formation. EpCAM has been recognized as an additional marker for CSCs in different tumor types (55). High expression of EpCAM in OC is associated with tumor recurrence, chemoresistance and poor patient prognosis (117). Cells co-expressing CD24, CD44, and EpCAM showed stem cell-like characteristics, high tumorigenicity *in vivo* as well as enhanced migration, invasion and colony-formation *in vitro* and this population could be enriched by chemotherapy (50, 118).

There are also several stem cell-associated genes that play a role in the maintenance and development of ovarian CSCs. *LIN28, OCT4, SOX2*, and *NANOG* transcription factors (TFs) regulate and maintain pluripotency in embryonic stem cells (119). The co-expression of *LIN28* and *OCT4* identifies a population of ovarian CSCs and correlates with advanced tumor grade (120). *NANOG* is closely associated with HGSC, high chemoresistance and poor overall patient survival (121). *SOX2* is required to maintain ovarian CSCs and its expression correlates with chemoresistance and poor prognosis (122).

### Mechanisms of Cancer Stem Cell Therapy Resistance

**Figure 2:**
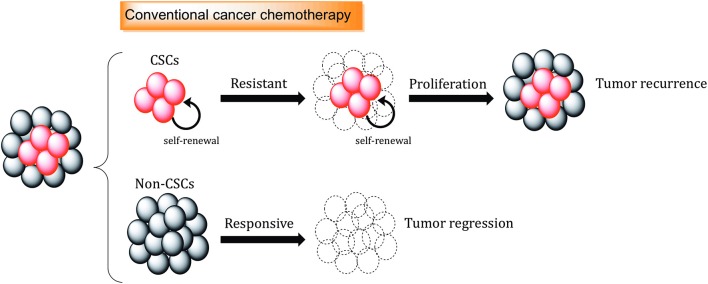
The role of cancer stem cells in drug resistance. Standard chemotherapeutics are not able to kill cancer stem cells (CSCs), which can promote tumor regrowth and eventually result in disease relapse. Different mechanisms, including increased.

Conventional therapies are often not sufficient to eliminate CSC populations because of intrinsic resistance mechanisms and epigenetic plasticity of the cells (123, 124). This means in many cases, tumor relapse is caused by the incomplete elimination of all CSCs, since a single CSC seems to be sufficient to regrow a tumor [Figure 2; (125)].

**Figure 3:**
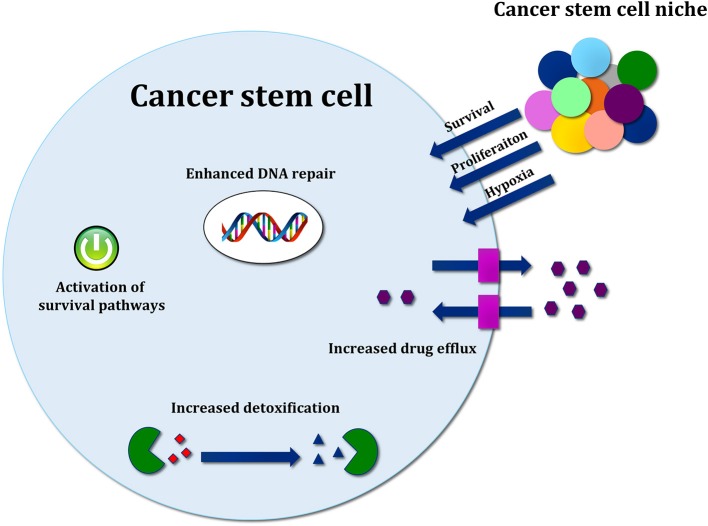
Mechanisms of cancer stem cell resistance toward conventional therapy. Therapy resistance mechanisms include drug efflux by ABC transporters, increased detoxification by ALDH activity, efficient ROS scavenging, and activation of developmental pathways mediating survival. Signals released by cancer stem cell (CSC) niche are also important to support survival, proliferation and therapy resistance.

Several molecular mechanisms, such as enhancement of DNA repair and DNA damage response (DDR), increased drug efflux, efficient scavenging of reactive oxygen species (ROS) and signals from the microenvironment, support the resistance of CSCs toward conventional cancer therapies [Figure 3; (126)].

### Increased DNA Pepaire Capacity

Radiation and many chemotherapeutic drugs (e.g., DNA crosslinkers, DNA synthesis, and topoisomerase inhibitors) induce DNA damage, which, if not repaired, can lead to cell death (127). Among different types of DNA damages caused by radiation and chemotherapy, DSBs are the most lethal lesions if not repaired (128). DSBs are usually repaired by error-free homologous recombination (HR) or error-prone non-homologous end joining (NHEJ) mechanisms (129). In response to radiation, DNA repair is initiated and controlled by the DNA damage response (DDR). For the purpose of stalling cell cycle progression and give time to the cells to repair DNA damage, DDR triggers the activation of checkpoint kinase signaling pathways such as ataxia telangiectasia mutated (ATM)-checkpoint kinase 2 (Chk2) and ATM-Rad3-related (ATR)-checkpoint kinase (Chk1) (130, 131). CSC populations of glioblastoma, prostate, lung, breast cancer and many others, have been shown to possess high DNA repair capacity, mainly due to increased activation of ATR-Chk1 and ATM-Chk2 pathways (130). Recent reports demonstrated increased HR-dependent DNA repair proficiency of CSCs in OC that makes them more resistant to PARP inhibition (132). Different populations of OC CSCs have a high resistance to the platinum agents due to the altered regulation of cell cycle checkpoint, upregulation of the Fanconi Anemia DNA repair proteins (*FANCD2, FANCJ*) (133), *MLH1*, and *BRCA1* (134) and increased expression of DNA polymerase eta (135).

### ROS Scavenging

Radiation-induced damages include the formation of ROS, which are chemically reactive free radicals produced by oxygen metabolism. ROS are involved in many physiological signaling pathways regulating metabolism, cell proliferation, migration, angiogenesis and wound healing (136). However, in high amounts, ROS can produce oxidative DNA damage, alter proteins and cell membrane lipid bilayer, leading to cell arrest and cell death (137). The cells control ROS levels through ROS scavenging molecules (e.g., glutathione peroxidase, superoxide dismutase, and catalase), which balance the production and the elimination of these products (137). CSCs isolated from different tumors exhibit more efficient ROS scavenging systems and a lower level of ROS production compared to non-CSC populations (138, 139). ALDH positive population in OC has an upregulation of NRF2 (Nuclear factor erythroid 2-like) signaling driving the cytoprotective response to the oxidative stress (140).

### Enhanced Drug Efflux by ABC Transporters

Another important mechanism for CSC drug resistance is the high expression of ATP-binding cassette (ABC) transporters. The human genome encodes 49 *ABC* genes and these proteins are essential to maintain homeostasis and to protect the cells against environmental insults in many normal tissues (e.g., liver, intestine, blood-brain barrier, placenta, kidney, and normal stem cells) (141). Three ABC transporter proteins, P-glycoprotein (P-gp, MDR1, ABCB1), multidrug resistance protein 1 (MRP1, ABCC1), and breast cancer resistance protein (BCRP, ABCG2), have been shown to play a role in multidrug resistance of various types of cancers like OC, breast, lung, colon and others (142). Due to broad substrate spectrum specificity, their expression provides tumor resistance toward the major classes of chemotherapeutic drugs (126). CSC populations of different tumors, including breast cancer, lung cancer, retinoblastoma, neuroblastoma, and glioblastoma, have been demonstrated to overexpress ABC transporters, which correlates with high drug resistance (143).

### Aldehyde Dehydrogenase Activity and Activation of Developmental Pathways

Supplementary intrinsic mechanisms underlying CSC therapy resistance are mediated by high ALDH activity and activation of developmental pathways essential for embryonic development and tissue homeostasis like the canonical Wnt/β-catenin, Notch and Hedgehog pathways (126). Notch signaling contributes to survival and platinum resistance of ovarian CSCs and it has been determined that Notch 3 expression is associated with poor prognosis for OC patients (144). Hedgehog signaling is aberrantly activated in OC and this pathway affects cell growth, motility, invasion, and tumorigenesis (145). Wnt/β-catenin is involved in stem cell proliferation and differentiation. CSCs of many tumors were found to overexpress β-catenin, which promotes stemness (146). In OC, genetic mutations in the Wnt pathway are rare, but recent data demonstrated that the activation of Wnt signaling could be regulated by the tumor microenvironment, contributing to ovarian tumorigenesis (147).

### Cancer Stem Cell Niche and Their Microenvironment

**Figure 4:**
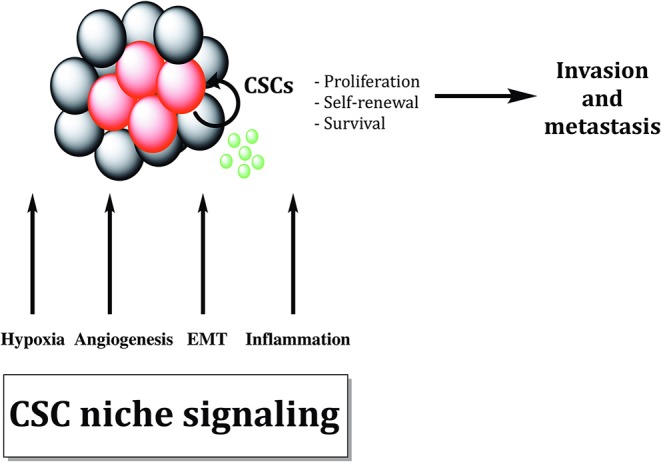
Cancer stem cells are sustained by biological processes within the cancer stem cell niche. Different biological processes like EMT, hypoxia, inflammation, and angiogenesis maintain cancer stem cells (CSCs). Cytokines and growth factors produced by tumor cells and tumor-associated cells increase proliferation and survival of CSCs, promoting tumor invasion and metastasis formation.

Growing bodies of evidence showed that microenvironmental stimuli coming from the specific niche in which CSCs reside could influence and regulate treatment responsiveness (Figure 4). Additionally, autocrine signaling and stimuli coming from stromal fibroblasts, immune cells and extracellular matrix (ECM) as well as oxygen, nutrient supply and tissue pH, might affect CSC properties and metastatic dissemination (136).

Matrix metalloproteinases (MMPs) are extracellular proteases able to degrade and cleave ECM molecules, remodeling CSC niche and releasing growth factors and cytokines, thus, being suggested to regulate signaling pathways that control proliferation, differentiation and tumor invasion (148).

Tumor and stroma-derived growth factors and cytokine regulating stemness and resistance comprise interleukin-6 (IL-6), interleukin-8 (IL-8), chemokine (C-X-C motif) ligand 12 (CXCL12), chemokine (C-X-C motif) receptor type 4 (CXCR4), chemokine (C-C motif) ligand 2 (CCL2), platelet-derived growth factor (PDGF), transforming growth factor-beta 1 (TGF-β1), tumor necrosis factor-alpha (TNFα), epidermal growth factor (EGF), VEGF and fibroblast growth factor (FGF) (62, 126). IL-6, which is secreted by tumor-associated stroma has been shown to play an important role in the enrichment of OC stem cells after treatment with platinum agents (149). Dual expression of CXCR4 and CD133 has been used to identify an OC population with stem cell-like properties that regulate tumor development and chemoresistance (92). The formation of new vessels, also known as angiogenesis, supports tumor growth and it is tightly regulated by angiogenic activators including VEGF, FGF, PDGF, and EGF (150). Bao et al. determined that glioblastoma CD133+ CSCs highly express VEGFA, which contributes to the angiogenic process by interacting with the microenvironment (151). A recent study revealed that VEGFA stimulates OC stem cells by activation of the epigenetic mechanisms inducing loss of miR128-2 and upregulation of Bmi1 (152).

The association between inflammation and cancer development has been suggested for numerous types of tumors (153). The accumulation of proliferating tumor cells mimics a chronic inflammation and the activated stroma cells produce a number of cytokines, angiogenic factors and chemokines, which in turn recruit more immune cells including monocytes and macrophages. The most common inflammatory molecules released in the microenvironment by tumor-associated macrophages (TAMs) are the EMT-inducers TGF-β1 and TNFα (154). EMT confers stem cell-like phenotype to cancer cells, enhancing motility and drug resistance.

In addition, tissue oxygenation influence CSC properties, maintaining cells in a quiescent (hypoxia) or activated (normoxia) state. For example, hypoxia is a major stimulant of CXCL12, which is secreted by tumor stroma fibroblasts and is involved in the early stage of malignant transformation of OC (139). Hypoxia makes CSCs more resistant to various environmental insults and it is the major stimulant of angiogenesis. Hypoxia-inducible factor (HIF) target genes, such as *GLUT-1, VEGF, OCT4* and Notch, are crucial regulating proliferation, tumorigenicity and self-renewal in different cancers (155). HIF induces stem cell properties in OC cells (156) and promotes OC stem cells adaptive stress response (157) and resistance to therapy (158). Hypoxia has been demonstrated to promote the acquisition of stem cell-like state through the increased expression of CSC markers like CD133 and CD44, along with CSC-related genes such as *SOX2* (155). Moreover, hypoxia is also tightly associated with chemo- and radioresistance. Low oxygen conditions maintain CSCs in a quiescent state with a low proliferation rate and since most conventional cytotoxic drugs target proliferating cells, CSCs cannot be destroyed. It is also recognized that the local concentration of oxygen improves radiotherapy efficacy since DNA lesions caused by ROS react with oxygen-generating stable DNA peroxides (159). Tumor oxygenation and oxygen therapeutics have been utilized as radiosensitizers to improve patients' response to radiotherapy (160).

### Epithelial-To-Mesenchymal Transition and Stemness

**Figure 5:**
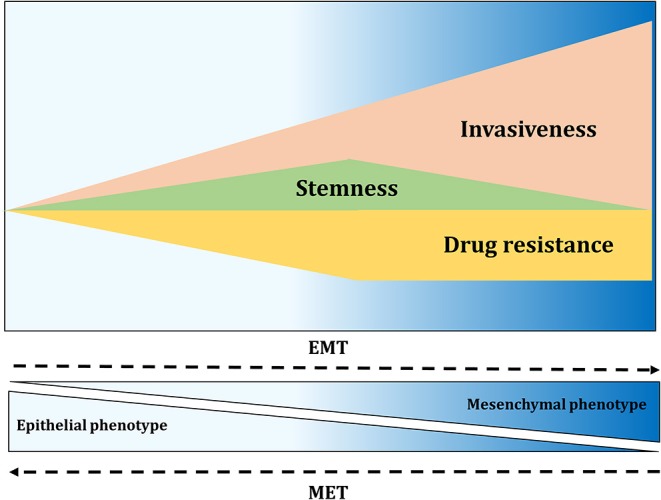
EMT contributes to invasiveness, stemness, and drug resistance. Activation of EMT results in migration and fast tissue invasion. Furthermore, EMT activation affects tumor-initiating properties of cancer cells, with the greatest properties found at the intermediate level of EMT. Drug resistance of cancer cells also seems to be highest in cells with intermediate EMT phenotype.

In recent years, high plasticity of CSCs and stemness have been increasingly linked to EMT. EMT is an essential cellular process usually involved in embryogenesis, wound healing and tumorigenesis. During tumor progression, neoplastic cells switch from an epithelial-like state to gain mesenchymal properties. Therefore, EMT allows cancer cells to become migratory and invasive, acquiring multiple properties associated with high-grade malignancies (161). EMT is suggested to be a reversible process where mesenchymal-like cells might transition back into an epithelial state, a process called mesenchymal-to-epithelial transition (MET). A number of TFs, referred as EMT-TFs, are associated with EMT and can be classified into three main protein families: Snail (Snail and Slug), ZEB (ZEB1 and ZEB2), and basic helix-loop-helix (TWIST1, TWIST2, and TCF3) families (162). These TFs coordinate changes in gene expression resulting in suppression of genes associated with the epithelial state, like E-cadherin, and in upregulation of genes correlated with the mesenchymal state, including N-cadherin and vimentin (162). The activation of some of these TFs, including Slug, ZEB1, ZEB2, TWIST1, and Snail1, has been linked to the expression of stem cell-related genes and self-renewal in various tumor types (163). The EMT process is controlled by a variety of cytokines and growth factors and among them TGF-β signaling plays a fundamental role. TGF-β signaling regulates ovarian CSCs through the modulation of tissue transglutaminase 2 (TGM2), which promotes EMT, metastasis and stem cell-like phenotype (164, 165). Hypoxia, through the expression of HIF-1α and HIF-2α, regulates CSC-associated genes and induces EMT via the TGF-β signaling pathway, which in turn contributes to stemness (166). Cancer cells with an intermediate EMT phenotype display stem cell-like properties accompanied by enhanced resistance to several anti-cancer drugs [Figure 5; (124)]. In 2011, Strauss and colleagues identified a minor population of OC cells in a transitory epithelial/mesenchymal hybrid stage (i.e., partial or intermediate EMT), which means that these cells expressed at the same time epithelial and mesenchymal markers, respectively linked to adhesion and migration (167). The observation that these cells drive tumor growth *in vivo* and have the capacity of self-renewal provided a link between stemness and phenotypic plasticity. Many other studies have highlighted a connection between partial EMT, drug resistance and tumor-initiating ability, showing that this phenotype enables the formation of highly metastatic circulating-tumor cells clusters (168). Therefore, EMT gives a hint about the plasticity model, showing that plasticity enhances invasion potential and tumor-initiating properties of cells that arrive at the metastatic site (169). Stemness is therefore not a fixed and inherent property but instead, CSCs and non-CSCs can bidirectionally interconvert between these two states (168).

## Novel Therapeutic Strategies for Ovarian Cancer

High-throughput approaches have identified numerous potential therapeutic targets to treat cancer cells specifically. The current focus of anti-cancer drug discovery is the development of molecular targeted therapies rather than conventional cytotoxic drugs. A major challenge remains to find agents and strategies that select and eliminate the source of tumor recurrence after therapy, along with bulk tumor cells. It became increasingly evident that CSCs are crucial for disease recurrence and chemoresistance, thus targeting these cells seems a promising way to reach complete tumor eradication.

### Inhibition of Cancer Stem Cell-Associated Pathways

Novel therapies have been employed to target CSC-specific markers or pathways critical for regulation and maintenance of stem cell-like properties (Table 2).

**Table 2:** Emerging therapies targeting ovarian cancer stem cell-associated pathways.

**Drug**	**Target**	**Identifier**	**Phase**	**Condition**	**Combination**
Metformin	Anti-diabetic drug	NCT02122185	II	Advanced OC	Yes
Sonidegib	Hedgehog inhibitor	NCT01954355	I	Advanced OC	Yes
Vismodegib	Hedgehog inhibitor	NCT00739661	II	OC in second or third complete remission	No
Ipafricept	Wnt inhibitor	NCT02092363	I	Recurrent platinum-sensitive OC	Yes
Defactinib	FAK inhibitor	NCT01778803	I	Advanced OC	Yes

*Clinical trial information accessed via https://clinicaltrials.gov with National Clinical Trial Number (NCT Number)*.

Metformin hydrochloride is clinically used as an anti-diabetic drug for type 2 diabetes. Recent studies have reported its ability to enhance chemotherapy efficacy in different cancer types by targeting CSCs. Metformin showed a synergic effect with standard chemotherapeutic agents, reducing tumor relapse rate (170). Shank and colleagues elucidated a potential mechanism behind this effect, demonstrating that the treatment with metformin reduced ALDH^+^ ovarian CSCs, proliferation and angiogenesis *in vitro* and *in vivo*. This effect was additive in combination with CDDP (171). The outcomes of combinational treatment with metformin and chemotherapy in patients with stage III and IV OC are currently investigated in phase II clinical trial (NCT02122185) (172). Treatment of OC cell lines with the FDA approved PARPi olaparib and rucaparib enriched cells with highly efficient DNA repair mechanisms. These cells expressed the CSC markers CD133 and CD117 (132). This might explain why patients who received PARPi treatment develop resistance in spite of a good initial therapy response. Several developmental pathways, including Notch, Wnt and Hedgehog, are crucial for self-renewal and regulation of CSCs (173). The inhibition of these pathways in combination with traditional chemotherapy has been proposed as a promising therapeutic strategy for recurrent diseases.

Sonidegib is an FDA approved Hedgehog inhibitor for basal cell carcinoma. Sonidegib was tested in phase I clinical trial in combination with PTX (NCT01954355) as a treatment for patients with advanced OC, demonstrating anti-tumoral activity. Based on this study, a recommended dose for phase II trial has been identified (174). The second Hedgehog inhibitor, Vismodegib, was used in phase II clinical trial as maintenance therapy for patients diagnosed with OC in second or third complete remission (NCT00739661) (175). However, no significant survival benefit was observed (5.8 months for placebo vs. 7.5 months for the treatment group), implying that the blockage of the Hedgehog pathway alone is probably not enough to destroy recurrent disease (175). The Wnt inhibitor Ipafricept reduces CSC frequency, promotes cell differentiation in patient-derived OC xenografts and in phase I clinical trial in association with carboplatin and PTX for recurrent platinum-sensitive OC (NCT02092363), 82% of patients achieved a partial or complete response (176). Focal adhesion kinase (FAK) is a cytoplasmic protein overexpressed in CSCs of different tumors and it maintains interaction with stromal cells to activate intracellular signaling cascades (62). Defactinib, an oral inhibitor of FAK, exhibited modest activity in phase I clinical trial in combination with PTX for advanced OC (NCT01778803), suggesting that targeting the CSC niche might offer a suitable therapy strategy (177).

### New Radioimmunotherapy Approaches

At the Center for Radiopharmaceutical Sciences of the Paul Scherrer Institute we developed the anti-L1 cell adhesion molecule (L1CAM) chimeric monoclonal antibody chCE7 for radioimmunotherapeutic (RIT) approaches to cancer. The L1CAM is a 200–220 kDa type I membrane glycoprotein of the immunoglobulin superfamily, containing six immunoglobulin-like domains and five fibronectin type III repeats followed by a transmembrane region and a highly conserved cytoplasmic tail (178–180). Firstly, L1CAM was identified as a protein of the nervous system and during brain development it was shown to play an important role in morphogenic events like neurite outgrowth, fasciculation, adhesion and neuronal cell migration (181). L1CAM is expressed in human peripheral nerve bundles and human kidneys, while it is absent in other human tissues comprising heart, lung, colon, liver, thymus or testis (181, 182). Low levels of L1CAM are also detectable in hematopoietic cells (181). L1CAM is overexpressed in various types of human cancers, including ovarian and endometrial carcinoma, colon cancer, melanoma and glioblastoma, and it usually correlates with advanced tumor stage and poor prognosis (179, 180). L1CAM expression in cancer supports motility and invasion, promoting aggressive tumor growth and metastasis formation (180). Additionally, L1CAM in combination with CD133 characterized a new ovarian CSC population (82). This protein exhibits static and motility-promoting functions, eliciting different signaling pathways depending on the binding partners; L1CAM homophilic interactions support static cell-cell binding while binding of L1CAM to integrin induces motility and invasiveness (180).

Indeed, the restricted expression of L1CAM allowed the use of anti-L1CAM mAbs for OC targeted therapies (183). Previous work revealed that chCE7 binds near the RGD sequence in the sixth Ig-like domain of human L1CAM, preventing its binding with integrin (184). RIT using copper-67 and the radiolanthanides lutetium-177 (^177^Lu) and terbium-161 (^161^Tb) was demonstrated to increase the efficiency of antibody-based L1CAM therapy in preclinical OC models (183, 185, 186). The antigen-antibody complex usually internalizes by endocytosis and the coupled metallic radionuclides are trapped in the cell (187). Therapeutic results for RIT in solid tumors (as adjuvant therapy after primary surgery and/or chemotherapy) have been modest during recent years and the main obstacles include low radiosensitivity, poor lymphatic drainage, limited diffusion of the antibody through tumor mass, poor vascularization and lack of homogeneous targeting (188). To overcome these limitations, several new strategies, including bioengineering development of different antibody formats to improve tumor penetration, pre-targeting approaches, the use of alpha particle or Auger electron emitters, as well as dose fractionation, have been developed over the past years (188). Furthermore, RIT has been combined with other chemotherapeutics, including DNA-damaging agents (e.g., CDDP), microtubules-stabilizing agents (e.g., PTX) and protein kinase inhibitors (PKIs) providing cellular radiosensitization effects (189, 190). PTX enhances anti-L1CAM RIT effectiveness, blocking cancer cells in the radiosensitive G2/M cell cycle phase. Our group could recently demonstrate that *in vivo* combination therapy with ^177^Lu-DOTA-chCE7 [^177^Lu was coupled to the mAb via the chelator 1,4,7,10-tetraazacyclododecane-N-N'-N”-N”'-tetraacetic acid (DOTA)] and PTX significantly extended overall survival of ovarian tumor-bearing mice (55 days RIT+PTX vs. 18 days PTX and 29 days RIT) (189).

Different properties of alpha-particles and Auger electrons, including short path length and high density of released energy in tissue, make them appropriate for targeted therapies against small volume diseases like metastases (<1 cm diameter) and for eradicating radioresistant CSCs (191).

**Figure 6:**
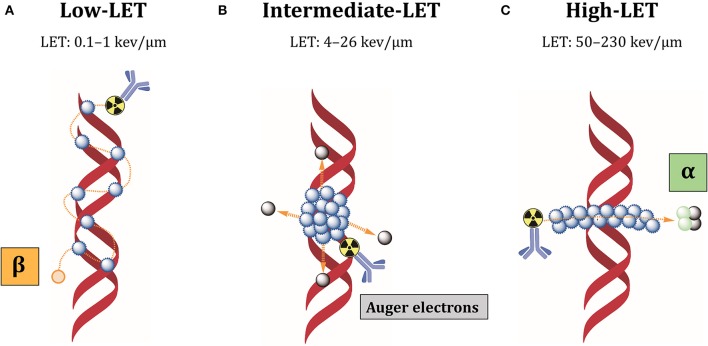
Cellular damages induced by different LET radiations. **(A)** Low-LET radiations produced by beta-particles induce individual DNA lesions that can be easily repaired by the DNA repair machinery. **(B)** Auger electrons are released as a cascade close to cell membrane or DNA, producing clusters of lethal DNA lesions. **(C)** Alpha radiations have high-LET and produce multiple damages along a linear track that are difficult to repair. Blue dots: ionizations/excitations.

The low-linear energy transfer (LET) refers to the energy released over the track length of the radiation in biological tissues. At equal absorbed dose, high-LET particles result in relatively high deposition of energy in tumor cells, which renders these forms of radiation more cytotoxic than low-LET radiations (Figure 6).

Most beta-particles emit low-LET radiations of 0.1–1 keV/μm, while alpha-particles have high-LET values of 50–230 keV/μm and Auger electrons intermediate-LET of 4 to 26 keV/μm (192). Low-LET of beta-particle emitters is due to intermediate energy (0.5–2.3 MeV) and long-range in biological tissues (0.5–12 mm), producing easily reparable sparse DNA lesions (193). Additionally, beta-particles kill tumor cells surrounding target cells independently from antigen expression, by the so-called “cross-fire” effect. Because of heterogeneous antigen expression among tumor cells, cross-fire irradiation is suitable when not all tumor cells can be specifically targeted by radiolabeled antibodies. However, the long path of these particles can also induce bone marrow toxicity. In contrast, alpha-particles have high energy (5–9 MeV) and intermediate path length (50–100 μm), which restricts their effect to 5–10 cell diameters (193). Auger electrons have low energy (0.001–1 KeV) and subcellular nanometer range (<1 μm) (193). These characteristics minimize the non-specific irradiation of non-targeted surrounding healthy tissues. Most importantly, it makes it possible to locally deliver high-absorbed doses and lethally damage tumor cells, inducing densely localized DNA DSBs. Besides DNA damage, these short-range electrons damage the cell membrane and elicit radiation-induced bystander effect (194). For this reason, alpha-particle and Auger electron-emitting nuclides are very attractive for RIT against therapy-resistant CSCs. Our group could recently demonstrate that L1CAM, combined with CD133, defines a new ovarian CSC population and L1CAM is responsible for the radioresistance of these cells (82). This cell population showed high spherogenic and clonogenic capabilities and highly expressed some CSC- and EMT-related genes. Additionally, these cells demonstrated high tumorigenicity, fast-tumor growth and self-renewal when transplanted in nude mice. CRISPR-*Cas9* deletion of L1CAM demonstrated that L1CAM expression correlates with EMT phenotype. Since L1CAM is not expressed in normal stem cells and it is present in the bulk population of cancer cells, anti-L1CAM RIT using Auger electrons and alpha-particle emitters would be a promising new option for ovarian CSCs therapy. In this context, we could clearly demonstrate in a comparative anti-L1CAM RIT study using ^177^Lu- and^161^Tb-labeled mAb chCE7 that ^161^Tb was better by 82.6% compared to ^177^Lu under equitoxic conditions in a preclinical OC xenograft model (186). ^161^Tb emits 16 times more Auger and conversion electrons per decay (3–50 keV) than ^177^Lu. Both radiolanthanides have similar ß–energy of 134 keV (^177^Lu; mean) or 154 keV (^161^Tb; mean). Data were taken from “National Nuclear Data Centre Brookhaven National Laboratory.”

Some RIT approaches for OC using alpha-particle emitters showed their great potential to treat microresidual diseases in preclinical models (194, 195). In 2017, Kasten et al. used the mAb 376.96, which recognizes the B7-H3 epitope expressed on ovarian CSCs and on the bulk population, radiolabeled with lead-212 (^212^Pb) to treat tumor-bearing mice. Mice treated with ^212^Pb-376.96, alone or combined with carboplatin, showed two to three times longer survival than control groups (195). Recent outcomes from a phase I clinical study indicated minimal toxicity of intraperitoneal (i.p) administration of ^212^Pb-TCMC-trastuzumab in patients with advanced OC (196).

## Conclusions

**Figure 7:**
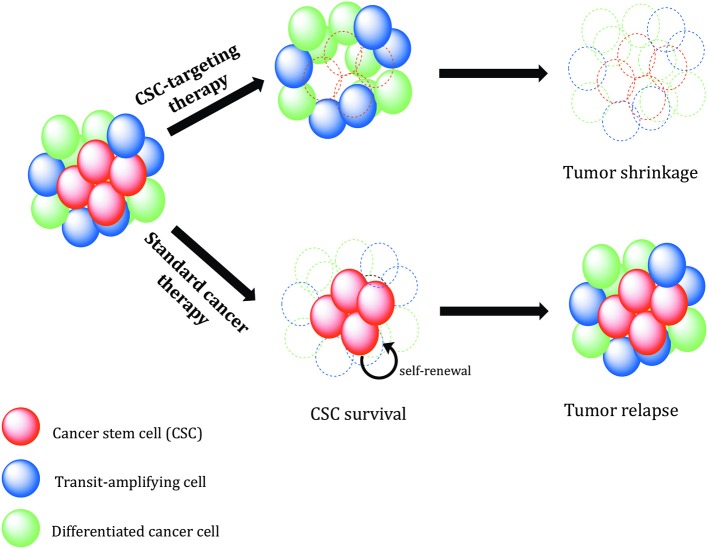
Therapeutic relevance of cancer stem cells. Conventional cancer therapies mostly kill the tumor bulk population, while therapy-resistant cancer stem cells (CSCs) survive and continue to proliferate, leading to tumor recurrence. CSC-specific therapies need to be designed in order to precisely eradicate CSCs. Without self-renewing CSCs to drive tumor progression, the tumor shrinks, and regresses.

The CSC heterogeneity and plasticity of CSC state is currently the largest obstacle to move CSC research toward clinical translation. Nevertheless, although the cancer stemness is now considered as a dynamic state rather than an entity, CSC-related biomarkers might be a powerful tool for prediction of patients' clinical outcomes, and targeting of CSC OC population might prove beneficial for the treatment of this deadly disease. Common knowledge about ovarian CSCs has made remarkable progress over the last years; still, there are many limitations and challenges related to disease complexity and current experimental techniques. Future studies are expected to employ clinically relevant models such as patient-derived xenografts, orthotopic mice models, and organoid cultures. Genetically engineered mice models provide opportunity to trace and validate potential CSC populations in the context of a fully functional immune system and tumor stroma. The inherent heterogeneity of OC provides therapeutic challenges and it is further complicated by the acquired heterogeneity driven by microenvironmental and therapeutic pressure. Clinical trials should consider the high heterogeneity regarding CSC markers to select the proper patient cohort. CSC frequency and content should be monitored during clinical trials to assess therapy response. Considering the possibility of bulk tumor cell reprogramming, an optimal therapeutic regimen should combine therapeutic drugs showing wide cytotoxic effects on non-CSCs in combination and/or followed by a therapy targeting resistant CSCs (Figure 7) (197). To reach this goal, research needs to focus on the identification of new and reliable signaling pathways that influence CSC maintenance, differentiation, drug resistance, DNA damage repair, and their plasticity. In addition, it would be helpful to identify new CSC cell surface markers that are not expressed in normal stem cells but are also present in the bulk population of tumor cells, like L1CAM in ovarian CSCs. In recent years, there has been a veritable renaissance of radiotherapeutic approaches and new promising radioisotopes were introduced in pre- and clinical trials. Especially alpha- and Auger-emitters allow the sterilization of radioresistant cells such as cancer stem cells due to their high-LET.

## Author Contributions

NT and JG designed the manuscript. NT wrote the first draft and illustrated the manuscript. NT, JG, FJ, and AD edited and reviewed the manuscript.

### Conflict of Interest

The authors declare that the research was conducted in the absence of any commercial or financial relationships that could be construed as a potential conflict of interest.
